# La maladie à IgG4: à propos de 3 cas

**DOI:** 10.11604/pamj.2020.36.364.24835

**Published:** 2020-08-28

**Authors:** Hakima Abid, Moulaye El Hacen Horma Babana El Alaoui, Moulay Youssef Alaoui Lamrani, Mouna Figuigui, Beiba Cheikh Ahmed, Nada Lahmidani, Mounia El Yousfi, Dafr-Allah Benajah, Mustapha Maaroufi, Mohammed El Abkari, Sidi Adil Ibrahimi, Nourdin Aqodad

**Affiliations:** 1 Service d´Hépato-Gastro-Entérologie, Centre Hospitalier Universitaire Hassan II, Fès, Maroc,; 2 Service de Radiologie, Centre Hospitalier Universitaire Hassan II, Fès, Maroc,; 3 Faculté de Médecine et de Pharmacie, Université Sidi Mohammed Ben Abdellah, Fès, Maroc; 4 Service d´Hépato-Gastro-Entérologie, Centre Hospitalier Universitaire Ibn Zohr, Agadir, Maroc

**Keywords:** IgG4, PAI type 1, corticothérapie, IgG4-related disease, PAI-1, corticosteroid therapy

## Abstract

La maladie à IgG4 encore appelée polyexocrinopathie auto-immune à IgG4 est une nouvelle entité où s´inscrit la PAI de type 1. Elle peut toucher différents organes (système nerveux central, les glandes salivaires, la thyroïde, les poumons, le pancréas, les voies biliaires, le foie, le tube digestif, les reins, la prostate…) avec des symptômes relatifs à l´organe atteint. Elle est plus fréquente chez l´homme de plus de 50 ans. Son incidence et sa prévalence sont mal connues car c´est une maladie peu fréquente. Elle est a priori plus fréquente en Asie et ne représente que 20 à 30% des PAI en occident. Son diagnostic est histologique caractérisé par la présence, dans un contexte d´élévation sérique des IgG4 dans plus de 80% des cas, d´un infiltrat lymphoplasmocytaire dense de l´organe atteint avec positivité des IgG4 en immuno- histochimie, d´une fibrose d´organe et des veinulites oblitérantes. Elle est sensible à la corticothérapie avec un risque de rechute à l´arrêt de la corticothérapie qui n´est pas négligeable et qui conduit alors à l´utilisation d´immunomodulateurs, principalement: les thiopurines (Azathioprine ou le 6-mercaptopurine), le méthotrexate et de façon plus récente le rituximab qui peut être utilisé également comme traitement d´induction. Grâce aux avancées récentes, des critères histologiques et cliniques précis sont maintenant connus permettant de limiter les prises en charge inadaptées telle que la chirurgie. Cependant, de nombreuses lacunes persistent dans nos connaissances: sur la physiopathologie, l´identification de biomarqueurs spécifiques autres que les IgG4, l´histoire naturelle de la maladie et l´évaluation du risque de cancer à long terme, les performances des outils diagnostiques comme la biopsie pancréatique sous échoendoscopie. De même, une prise en charge consensuelle internationale reste à définir dans les phases initiales de la maladie et en cas de rechute. L´objectif de cette étude est de rapporter 3 cas de ML-IgG4 en se basant sur les critères cliniques et radiologiques, la réponse thérapeutique.

## Abstract (en)

IgG4-Related disease (IgG4-RD), formerly known as IgG4-related autoimmune polyexocrinopathy, is a new condition including Plasminogen Activator Inhibitor-1 (PAI-1). It can affect different organs (central nervous system, salivary glands, thyroid, lungs, pancreas, bile ducts, liver, digestive tract, kidneys, prostate, etc.) with symptoms depending on the organ that is affected. It is more common in men older than 50 years of age. Its incidence and prevalence are poorly known because it is an uncommon disease. It is most common in Asia, accounting for only 20-30% of PAI in the Western world. Diagnosis is based on histological examination which shows dense lymphoplasmocytic infiltration in the organ affected associated with IgG4-positive plasma cells (immunohistochemistry), organ fibrosis and obliterating venulitis, all this in the context of increased serum IgG4 levels in more than 80% of cases. Patients are sensitive to corticosteroid therapy, with a high risk of relapse after discontinuation of corticosteroid therapy. This leads to the use of immunomodulators, mainly: thiopurines (azathioprine or 6-mercaptopurine), methotrexate and more recently rituximab, which can also be used as induction therapy. Given recent advances, accurate histological and clinical criteria are currently known to limit inappropriate management such as surgery. However, knowledge gaps remain concerning: pathophysiology, identification of specific biomarkers other than IgG4, natural history of the disease and long-term cancer risk assessment, performances of diagnostic tools such as endoscopic ultrasound-guided pancreatic biopsy. As well, consensual international management should be defined in the early stages of the disease and when patients develop recurrences. The purpose of this study was to report 3 cases of IgG4-Related disease on the basis of clinical and radiological criteria as well as therapeutic response.

## Introduction

La maladie à IgG4 (ML-IgG4), entité anatomoclinique reconnue sur le plan international sous le terme consensuel d´*IgG4-related disease*, est en fait une maladie ancienne identifiée par différents spécialistes d´organe sous de nombreuses dénominations (syndrome de Mikulicz, thyroïdite de Riedel, fibrose retro péritonéale). L´incidence et la prévalence de la maladie à IgG4 ne sont pas connues car c´est une maladie peu fréquente. De plus, les critères diagnostiques ont beaucoup évolué au cours des dernières années et les études ne sont pas homogènes. La majorité des études est issue de pays asiatiques (Japon et Corée du Sud) et sont focalisées sur la PAI. La prévalence de la PAI a été estimée à 6% au sein des pancréatites dite idiopathiques en Asie. Cette part est probablement sous-estimée par défaut diagnostique. Au Japon, la prévalence a été estimée à 0,82/100000 habitants. C´est au début des années 2000 que la maladie a été identifiée chez des patients présentant une pancréatite auto-immune (PAI) [1]. Par la suite la maladie associée aux IgG4 a été décrite comme pouvant toucher quasiment tous les organes.

Les principaux organes concernés sont le pancréas et les voies biliaires, les glandes salivaires et lacrymales, les ganglions médiastinaux et le rétro péritoine, les poumons, les reins, la pathologie inflammatoire aortique [2] (Tableau 1) [3]. Elle est plus fréquente chez l´homme de plus de 50 ans. Les manifestations cliniques sont le plus souvent subaiguës, sous forme d´atteintes lentement progressives d´un ou de plusieurs organes. Le diagnostic peut se faire de manière fortuite par la découverte d´anomalies radiologiques (mise en évidence d´adénopathies, par exemple) ou histopathologiques caractéristiques (Tableau 2) [4]. Elle est souvent associée à une élévation du taux des IgG4 sériques supérieur à 1,35g/L. L´aspect histopathologiques est celui d´une augmentation de volume parfois pseudo tumorale des organes atteints et ceci est dû à une infiltration lymphoplasmocytaire à prédominance de plasmocytes IgG4 positifs et d´une fibrose progressive et des lésions de thrombophlébite oblitérante (Tableau 3) [5]. Elle est sensible à la corticothérapie avec un risque de rechute à l´arrêt de la corticothérapie qui n´est pas négligeable et qui conduit alors à l´utilisation d´immunomodulateurs, principalement: les thiopurines (Azathioprine ou le 6-mercaptopurine), le méthotrexate et de façon plus récente le rituximab qui peut être utilisé également comme traitement d´induction [6]. L´objectif de cette étude est de rapporter 3 cas de ML-IgG4 en se basant sur les critères cliniques et radiologiques, la réponse thérapeutique.

**Tableau 1:** diverses maladies maintenant regroupées sous le nom de “maladie liée aux immunoglobulines de type G4 (IgG4)” (adapté de Perez et coll.)

Organe(s) atteint(s)	Nom de maladie
Aorte	- Anévrisme aortique inflammatoire
Artères	- Périaortite / périartérite
Glandes salivaires et lacrymales	- Maladie de Mikulicz
Glandes submandibulaires	- Tumeur de Küttner
Médiastin	- Médiastinite sclérosante
Méninges	- Pachyméningite hypertrophique idiopathique
Mésentère	- Mésentérite sclérosante
Organes multiples	- Fibrosclérose multifocale - Pseudotumeur inflammatoire
Pancréas	- Pancréatite sclérosante lymphoplasmocytaire - Pancréatite auto-immune
Peau	- Pseudolymphome cutanée
Région cervicale (tissu mou)	- Fibrose cervicale idiopathique
Reins	- Néphrite tubulo-interstitielle idiopathique (NTI) - NTI hypocomplémentémique avec dépôts tubulo-interstitiels extensifs
Rétropéritoine	- Fibrose rétropéritonéale
Sinus et cavité nasale	- Fibrose éosinophile angio-centrique
Thyroïde	- Thyroïdite de Riedel
Voies biliaires	- Cholangite sclérosante

**Tableau 2:** critères diagnostiques pour la maladie liées aux immunoglobulines de type G4 (IgG4)

Signes d´organe(s) diffusément ou partiellement agrandi(s), ou sous forme nodulaire
Taux sérique d´IgG4 > ou égal à 1.35g/l
**Examen histopathologique:** infiltrat lymphoplasmocytaire avec sclérose Ratio de cellules IgG4+ / IgG+ > 40%, et > ou égal 10 plasmocytes-IgG4+ / HPF
Diagnostic confirmé: 1+2+3; probable 1+3; possible 1+2 HPF: high power field

**Tableau 3:** caractéristiques histopathologiques de la maladie liée aux immunoglobulines de type G4 (IgG4)

Infiltrat lymphocytaire avec prédominance de plasmocytes IgG4 positifs et de lymphocytes T CD4+
Fibrose façonnée de manière " storiforme lrquo;"
Phlébites obstructives
Désigne un motif en roue de carasse avec un centre parfois spiralé, et des bandes de fibrose émanant de celui-ci avec une trame irrégulière emmêlée ressemblant un peu à celle d´une natte de paille.

## Étude de cas

### Observation n°1

Patient âgé de 63 ans, suivi pour diabète type II sous ADO avec notion d´alcoolisme chronique pendant plus de 15 ans sevré il y´a 10 ans; ayant comme motif d´hospitalisation une douleur de l´hypochondre droit irradiant à l´épaule droite associée à des vomissements. A l´examen clinique: patient sub-ictérique avec sensibilité épigastrique et de l´hypochondre droit. A la biologie on note une élévation de la CRP à 30mg/l avec une cholestase ictérique (bilirubine totale à 39mg/l à prédominance direct à 29mg/l, GGT à 1430 UI/l soit 26 fois la normale et PAL à 720UI/l soit 6 fois la normale) avec une cytolyse prédominante sur les ALAT à 175UI/l soit 3.5 fois la normale. L´échographie abdominale a mis en évidence une vésicule biliaire multilithiasique à paroi fine avec dilatation VBP à 9mm sans obstacle visible. Le scanner abdominale a montré une VB multilithiasique avec une VBP dilatée à 9mm, un pancréas augmenté de volume avec une infiltration autour du tronc cœliaque et l´AMS ainsi que l´aorte abdominale. Un bilan de 2^e^ intention comportant un bilan d´auto-immunité, phosphocalcique, lipidique et une pancréato-bili-IRM a été demandé avec comme résultats:

***Sérologies virales:*** B, C et E négatives; l´électrophorèse des protides normaux. Le bilan d´auto-immunité était revenu négatif avec des AAN négatifs, Ac anti-mitochondrie, anti-LKM1, anti-LC1 et anti-SLA négatifs. Le bilan lipidique avait retrouvé une hypercholestérolémie et une hypertriglycéridémie et le bilan phosphocalcique normal. L´ACE était élevé à 156 µg/L. le dosage de l´IgG4 était revenu> 6,8g/L. La pancréato-bili-IRM quant à elle avait mis en évidence une sténose de la VBP avec dilatation de sa partie moyenne avec dilatation VBIH ainsi qu´une fibrose retropéritonéale (Aorte et VMS). L´échoendoscopie avait mis en évidence une dilatation modérée des VBIH avec une VBP épaissie, non dilatée mesurant 7mm sans obstacle et un pancréas tuméfié très hétérogène dans son ensemble. Suite à ces données biologiques et radiologiques le diagnostic d´une CHOLANGITE à IgG4 (atteinte extra-pancréatique) + PAI type I a été retenu et le patient a été mis sous corticothérapie à la dose de 40mg/j avec surveillance de la glycémie. L´évolution a été favorable avec disparition de l´ictère à j15 avec normalisation du bilan hépatique à j20 et normalisation du taux IgG4 à j35.

### Observation n°2

Patient âgé de 50 ans, sans antécédents pathologiques notables admis pour prise en charge de douleurs abdominales diffuses atypiques, d´intensité modérée, évoluant depuis 5 mois associés à des vomissements intermittents. L´examen clinique ne retrouve qu´une sensibilité à la palpation de la région épigastrique. A la biologie, on notait une hyperoesinophilie avec normalité du bilan hépatique et des enzymes pancréatiques. L´échographie abdominale avait mis en évidence un très gros pancréas, hypoéchogène avec compression du tronc spléno-mésaraïque et au scanner abdominale l´aspect était celui d´un pancréas hypertrophique avec épaississement diffus entouré d´un halo qui reste hypodense au temps portal et quelques petites plages hypodenses caudales et périphériques rappelant un pancréas en saucisse. Il s´y associe une perte de lobulation avec un effacement du canal pancréatique principal. La face postérieure du pancréas infiltre la veine splénique, la rendant sténosée avec développement de plusieurs dérivations péri-gastriques et péri-spléniques + splénomégalie. Le bilan de 2^e^ intention avait mis en évidence une augmentation légère des gammaglobulines à 1,3 fois la normale avec une valeur d´IgG4 à 1,98g/l. Une fibroscopie œso-gastro-duodénale + echoendoscopie ont été réalisés avec comme résultats: gastrite antro-fundique érythémateuse et à l´échoendoscopie on retrouve un pancréas hétérogène hypertrophique surtout au niveau de la tête avec de multiples images hyperéchogènes avec des cônes d´ombres; compression de la veine mésentérique supérieure avec disparition du flux; visualisation du Wirsung au niveau de sa partie corporéo-caudale avec un aspect irrégulier et non dilaté. Un bilan de thrombophilie réalisé, suite à la découverte de cette thrombose de la VMS, est revenu normal.

***Suite à ces résultats:*** élévation des IgG4 ainsi que l´aspect radiologique, le diagnostic de pancréatite auto-immune à IgG4 a été retenu et le patient a été mis sous corticothérapie à la dose de 40 mg/j + HBPM qui fut par la suite switchée aux anticoagulants (Sintrom). L´évolution sous traitement était favorable avec disparition de la symptomatologie clinique ainsi qu´une normalisation du taux IgG4 à j17. Actuellement le patient a arrêté la corticothérapie depuis 5 mois sans rechute.

### Observation n°3

Patient âgé de 81 ans, suivi pour un asthme sous traitement, un diabète de type II sous ADO ainsi qu´un vitiligo et une hépatite C traitée et déclarée guéri, ayant comme motif d´hospitalisation un ictère et prurit. L´histoire de la maladie remonte à 3 mois par l´installation progressive d´un ictère avec urines foncées et selles décolorées ainsi qu´un prurit augmentant progressivement d´intensité. Le tout évoluant dans un contexte d´AEG avec perte de poids estimée à 20kg en 3 mois. A la biologie on notait une cholestase ictérique (Bilirubine totale à 89 mg/l à prédominance direct à 68mg/l, PAL 636UI/l et des GGT 535UI/l) ainsi qu´une légère cytolyse avec élévation des marqueurs tumoraux CA 19-9 à 166,4U/ml. A la bili-IRM on notait une hypertrophie de la tête du pancréas siège d´un processus tumoral mesurant 3,8cm x 3,1cm iso-intense en T1 et T2 et hypo-intense en diffusion avec envahissement vasculaire et rehaussement intense après injection et image d´arrêt du cholédoque ainsi qu´une dilatation des VBIH et VBP à 13mm ainsi qu´une grosse vésicule. Vu l´accentuation de l´ictère et l´augmentation de l´intensité du prurit, la patiente a été drainée par voie endoscopique avec comme constatation lors du geste d´une dilatation de la VBP en amont d´une sténose irrégulière de la portion rétro-pancréatique étendue sur 15mm avec mise en place d´une prothèse plastique assurant un bon drainage. En post-drainage le patient a amélioré ses chiffres de cholestase avec baisse de la Bilirubine totale à 20 mg/l. Une imagerie de contrôle par Angioscanner avait noté une nette régression de la dilatation bicanalaire et de la masse pancréatique avec un épaississement circonférentiel, régulier et symétrique de la VB et de la VBP ainsi que le canal cystique et présence d´une adénopathie rétropéritonéale sous rénale gauche de 10mm.

Suite à ceci, un bilan de 2^e^ intention comportant un bilan d´auto-immunité, phosphocalcique, lipidique, une échoendoscopie et une pancréato-bili-IRM ont été demandés avec comme résultats: les sérologies virales B et E négatives avec une charge virale VHC indétectable; l´électrophorèse des protides avait retrouvé un bloc béta-gamma. Le bilan d´auto-immunité était revenu négatif avec des AAN négatifs, Ac anti-mitochondrie, anti-LKM1, anti-LC1 et anti-SLA négatifs. Et le dosage des Immunoglobuline: IgA à 4,5g/l, les IgG à 23,75g/L et IgM à 1,61g/l. Le bilan lipidique avait retrouvé une hypertriglycéridémie et le bilan phosphocalcique normal. L´ACE à 6,68µg/L et le CA 19-9 à 246,4U/ml. Le dosage de l´IgG4 était revenu > 3,11g/L soit 3,6 fois la normale. A l´échoendoscopie on notait une dilatation des VBIH avec épaississement de la paroi de la VBP à 3mm dans toute sa longueur ainsi qu´une masse hypoéchogène hétérogène de la tête du pancréas mesurant 15mm; Aspect évoquant une cholangio-pancréatite auto-immune. La pancréato-IRM quant à elle a retrouvé une régression quasi-totale de la masse céphalique avec atrophie corporéo-caudale associée à une dilatation de la VBIH avec rehaussement de leurs parois en rapport avec une cholangite. Suite à ces données biologiques et radiologiques le diagnostic d´une PAI type I + CHOLANGITE à IgG4 a été retenu et le patient a été mis sous corticothérapie à la dose de 40mg/j avec surveillance de la glycémie. L´évolution a été favorable avec normalisation du bilan hépatique à j10 et du taux IgG4 à j30. Actuellement le patient et en cours de dégression de corticothérapie.

## Discussion

L´association entre pancréatite sclérosante (ou pancréatite auto-immune de type I) et taux élevé d´IgG4 a été décrite la première fois en 2001 [7]. Depuis lors, un nombre croissant de maladies inflammatoires a rejoint cette nouvelle entité, appelée *IgG4-related disease* ou «maladie liée aux IgG4» (MLIgG4) (Tableau 1) [3]. La ML-IgG4 rassemble donc des atteintes inflammatoires de divers organes autour d´une entité nosologique commune, de façon un peu analogue à la sarcoïdose. Les caractéristiques cliniques, paracliniques et histopathologiques de la ML-IgG4 ont fait l´objet d´un consensus international adopté en 2011 [4, 5]. Le terme ML-IgG4 devrait dès lors remplacer les autres terminologies anciennement utilisées pour les pathologies décrites dans le Tableau 1[3, 8]. Elle peut toucher un ou plusieurs organes de manière synchrone ou différée, sous forme de masse ou d´agrandissement diffus des organes atteints. L´aspect histopathologique est celui d´une infiltration lymphoplasmocytaire et d´une fibrose progressive. Les organes les plus souvent touchés sont le pancréas, les voies biliaires, les glandes salivaires, les glandes lacrymales, les ganglions médiastinaux, le rétropéritoine, l´aorte, les poumons et les reins [9]. Des atteintes d´organes multiples sont par ailleurs retrouvées dans 60 à 90% des cas [10, 11]. Les différents organes atteints et la présentation clinique de la ML-IgG4 ont été énumérés dans une excellente revue publiée en 2014 par Brito-Zerón et coll. [12]. Chez nos patients l´atteinte était surtout pancréatique, biliaire et retropéritonéale. Les manifestations cliniques sont le plus souvent subaiguës, sous forme d´atteintes lentement progressives d´un ou de plusieurs organes. Parfois, le patient est asymptomatique. Le diagnostic peut se faire de manière fortuite par la découverte d´anomalies radiologiques (mise en évidence d´adénopathies, par exemple) ou histopathologiques caractéristiques. Des anomalies de laboratoire de type allergique (élévation du taux sérique d´IgE totales, et du compte de cellules éosinophiles circulantes) peuvent être associées dans environ 40% des cas [13].

La PAI est la présentation clinique qui a permis de mieux comprendre et de décrire la ML-IgG4. La PAI de type 1 est très fréquente en Asie (>90% des séries japonaises) et représente moins de 20% des séries occidentales. Les patients sont âgés en moyenne de plus de 50 ans dans 80% des cas et sont de sexe masculin dans une large majorité des cas (80%) et ceci est le cas de nos patients. La présentation clinique peut être variée: soit liée à l´atteinte de la glande pancréatique: ictère par compression de la voie biliaire principale dans sa portion rétro-pancréatique, forme pseudotumorale, pancréatite aiguë (plus rare), insuffisance pancréatique exocrine ou endocrine ; soit liée à une atteinte extra-pancréatique fréquente (> 60-70% des cas): ictère par atteinte spécifique des voies biliaires, syndrome sec, fibrose retropéritonéale.

Dans notre étude, l'ictère était lié à une atteinte extra-pancréatique chez le premier patient et à une atteinte de la glande elle-même chez les deux autres. La forme pseudotumorale était retrouvée chez deux de nos patients. Chez le premier patient l´aspect mimait un cholangiocarcinome (CC) et chez le troisième patient une tumeur pancréatique. La probabilité d´avoir une ML-IgG4 augmente en cas de taux sérique d´IgG4 élevé, de symptômes allergiques, ou de processus fibrotique [14]. Outre l´examen clinique, le diagnostic repose donc sur des données paracliniques associant le dosage des immunoglobulines (IgG4 sériques ≥1,35g/l), l´imagerie et surtout l´aspect histopathologique des prélèvements tissulaires. Carruthers et coll. ont étudié l´utilité diagnostique du taux sérique d´IgG4 dans la ML-IgG4 [15]. Malgré une sensibilité de 90% et une valeur prédictive négative de 96% pour le diagnostic de ML-IgG4, sa spécificité (60%) et sa valeur prédictive positive (34%) sont faibles, du fait que d´autres conditions peuvent être associées à une augmentation des IgG4 [16]. Un seuil de 135mg/dL semble être utile pour distinguer les ML- IgG4 du cancer du pancréas et de la Cholangite sclérosante primitive (CSP). Cependant, il a une spécificité plus faible pour distinguer la ML-IgG4 du CC [17]. Ohara *et al*. [18] ont établi un seuil pour distinguer les ML- IgG4 des CC en utilisant les taux sériques mesurés dans neuf centres japonais. Un seuil de 182 mg/dl à une spécificité de 96,6% et un seuil de 207 mg/dl a une sensibilité et une spécificité plus élevées pour distinguer les CC de types 3 et 4 de la ML-IgG4. Nos trois patients avaient des taux sériques d´IgG4 dépassant ces deux seuils renforçant ainsi le diagnostic de ML-IgG4.

Les critères proposés pour le diagnostic de la ML-IgG4 sont présentés dans le Tableau 1 [3, 8]. La maladie est considérée comme confirmée, probable, ou possible selon le nombre de critères remplis. La présence de thrombophlébite oblitérante et d´éosinophilie est caractéristique mais inconstante et ne fait donc pas partie des critères diagnostiques (9). Chez nos trois patients les critères remplis étaient ceux biologiques par élévations des IgG4 et radiologique donc critères 1 + 2 du Tableau 1 rendant le diagnostic possible. La ML-IgG4 est une maladie très sensible à la corticothérapie. Ce traitement reste donc le traitement de première intention dans cette pathologie, même si la plupart des informations retenues l´ont été à partir d´études rétrospectives, en particulier dans les pancréatites auto-immunes. Les indications ainsi que le schéma thérapeutique ont été posés par l´International consensus for the treatment of autoimmune pancreatitis [6] qui définit quatre niveaux de recommandations: «fortement recommandable», (niveau A) ou «fortement non recommandable (niveau D)», et «normalement recommandable» (niveau B), «non recommandable» (niveau C) [6].

Le traitement est indiqué chez les patients symptomatiques avec atteinte pancréatique (par exemple, ictère obstructif , douleurs abdominales) ou autre atteinte d'organe (par exemple, ictère secondaire à un rétrécissement des voies biliaires) (niveau B) mais également chez les patients asymptomatiques mais présentant une masse pancréatique persistante à l´imagerie ou les patients ayant une cholangite à IgG4 avec persistance de la perturbation du bilan hépatique (niveau B) [6]. La corticothérapie constitue le pilier principal du traitement d´induction avec un taux de réponse très élevé [6] mais son schéma est variable selon les écoles et les auteurs japonais qui ont le plus d´expérience dans cette affection proposent le consensus de traitement suivant [6]: prednisolone à la posologie de 0,6 à 1mg/kg par jour pendant deux à quatre semaines. A l´issue de cette dose d´attaque, la posologie sera diminuée de 5 à 10 mg/jour toutes les 1 à 2 semaines jusqu'à une dose quotidienne de 20mg, suivie d'une réduction progressive de 5mg toutes les 2 semaines jusqu´à arrêt. La durée du traitement de rémission totale devrait généralement durer 12 semaines.

De nombreux experts japonais recommandent l´utilisation d´un traitement de maintien à base de glucocorticoïdes à faible dose (2,5 à 7,5mg/jour) pendant un maximum de 3 ans et l´arrêt du traitement de maintien doit être planifié dans les cas d´amélioration radiologique et sérologique [19]. Ce schéma thérapeutique a été instauré chez nos deux patients avec bonne amélioration clinico-biologique: disparition de l´ictère à j15 avec une normalisation du bilan hépatique à j20 et taux IgG4 à j35 dans le cas 1 et normalisation du taux IgG4 à j17 dans le cas 2. Avec ce schéma thérapeutique, environ 25% des patients présentent toutefois des reprises évolutives [20]. En cas de corticodépendance ou surtout en cas d´effets secondaires chez des patients déjà possiblement atteint d´ostéoporose ou de diabète, des immunosuppresseurs ou d´autres traitements peuvent être utilisés: Rituximab, Azathioprine (2 à 2,5mg/kg par jour), le Mycophénolate mofétil (750mg par jour) [6]. Ces derniers sont les deux immunosuppresseurs les plus utilisés. En raison du taux élevé de récidive, notamment en cas d´atteinte biliaire, une surveillance est recommandée. Les facteurs prédictifs de rechutes incluent un taux d'IgG4 sériques remarquablement élevés (tels que> x4 UNL) avant le traitement et persistance d´un taux toujours élevé après le traitement par les stéroïdes; la présence d´un élargissement diffus du pancréas, une cholangite proximale ainsi que l´atteinte de plusieurs organes [6]. La surveillance se fait comme suit: un bilan hépatique (transaminases, phosphatases alcalines et bilirubine totale/conjuguée) et dosage des IgG4 sériques tous les 3 mois pendant 2 ans et réalisation d´une IRM pancréatique et biliaire tous les ans pendant 2 ans. Nos trois patients étaient suivis suivant ce protocole avec comme résultats:

***Patients n°1:*** bilan à 3 mois normal, et celui à 6 mois revenant perturbé avec cholestase ictérique, cytolyse et ascension de l´ACE à 354 µg/L et à la pancréato-bili-IRM il y´avait une nette régression de la fibrose retropéritonéale avec disparition de la tuméfaction pancréatique cependant il y´avait une persistance de la sténose de la VBP et apparition d´une sténose du hile hépatique faisant suspecter un cholangiocarcinome. Devant ceci le patient a été remis sous corticothérapie 40mg/j pendant 1 mois. Un bilan hépatique, de contrôle, réalisé 15 jours après corticothérapie était revenu strictement normal et une bili-IRM à un mois a noté une disparition de la sténose hilaire avec régression de la sténose de la VBP. Le patient a été mis, par la suite, sous Immunosuppresseur (Azathioprine) avec bonne amélioration.

## Conclusion

La maladie à IgG4 est une nouvelle entité où s´inscrit la PAI de type 1. Elle est caractérisée par une atteinte multiorganique dont les particularités histologiques sont un infiltrat lymphoplasmocytaire (positif en immunohistochimie pour les IgG4) dense, une fibrose d´organe et des veinulites oblitérantes. Cette maladie auto-immune est dite systémique à IgG4 en raison de taux sériques fortement élevées. Elle doit être suspectée face à tout tableau clinique subaigu d´inflammation ou de fibrose avec une apparence radiologique pseudotumorale des organes impliqués. La suspicion est d´autant plus grande si le taux sérique d´IgG4 est augmenté. Le diagnostic est confirmé par un aspect histopathologique caractéristique des biopsies. Le processus physiopathologique de cette maladie n´est pas encore complètement élucidé et doit faire l´objet d´études approfondies, concernant notamment: le lien entre l´histopathologie, l´augmentation du taux sérique d´IgG4, et la quantité augmentée de plasmocytes-IgG4 positifs; le rôle des IgG4 dans la ML-IgG4 et la réponse aux différents traitements proposés.
